# Economic impact of using maternal plasma cell‐free DNA testing to guide further workup in recurrent pregnancy loss

**DOI:** 10.1002/pd.5972

**Published:** 2021-05-24

**Authors:** Siyang Peng, Sucheta Bhatt, Antoni Borrell, Yuval Yaron

**Affiliations:** ^1^ Illumina Inc. San Diego CA United States of America; ^2^ BCNatal Hospital Clinic Barcelona Catalonia Spain; ^3^ Institut d’Investigacions Biomediques August Pi i Sunyer (IDIBAPS) Barcelona Catalonia Spain; ^4^ Prenatal Genetic Diagnosis Unit Genetic Institute Tel Aviv Sourasky Medical Center Tel Aviv Israel; ^5^ Faculty of Medicine Tel Aviv University Tel Aviv Israel

## Abstract

**Objective:**

We have previously demonstrated that maternal‐plasma cell‐free DNA (cfDNA)‐testing can detect chromosomal anomalies in recurrent pregnancy loss (RPL) with 81.8% sensitivity and 90.3% specificity. Here we assess whether this is cost effective in guiding further workup in RPLs.

**Method:**

A decision‐analytic model was developed to compare the cost of various RPL management pathways: (1) current American Society for Reproductive Medicine (ASRM) RPL workup; (2) microarray or karyotyping analysis of products of conception (POCs) and RPL workup only for euploid cases; and (3) cfDNA testing and RPL workup only for euploid cases. Sample accessibility, failure rates, and sensitivity were specified for each test. Costs of sample collection, genetic tests, and RPL workup were considered. Analysis outcomes included detection rate of chromosomal anomaly and cost per patient tested.

**Results:**

In comparison to existing cytogenetic testing on POCs, cfDNA testing pathway allowed for better sample accessibility with a lower cost per patient. In addition, using cfDNA to guide further workup significantly increases the number of causative fetal chromosome anomalies detected, reducing the number of patients undergoing unnecessary workup resulting in an overall cost savings.

**Conclusion:**

Our study showed that inclusion of cfDNA testing is a cost‐effective approach to guide RPL workup.

## Key points

What is already known about this topic?

- Chromosomal anomalies account for 50%–70% of early pregnancy loss (EPL) and even in recurrent pregnancy loss (RPL) random aneuploidy is the single most common etiology, accounting for >50% of cases.
- In a previous study we have shown that maternal‐plasma genome‐wide cell‐free DNA (cfDNA)‐based testing can reliably detect chromosomal anomalies in random EPL and RPL with a sensitivity of 81.8% and a specificity of 90.3%.
- We therefore suggested cfDNA‐based testing serve to guide further management in cases of RPL: if cfDNA in the second and subsequent RPL demonstrates aneuploidy, no further action is taken; if an unbalanced rearrangement is found, parental karyotyping is recommended; if no abnormality is detected, the recommended RPL workup is performed.

What does this study add?

- In this study we demonstrate that using cfDNA to guide further workup in RPL is a cost‐effective approach.
- We share our algorithm to facilitate local cost‐effectiveness analysis based on prevailing billing schemes, either based on national health systems or private payor systems.

## 1 INTRODUCTION

Early pregnancy loss (EPL), defined as a nonviable, intrauterine pregnancy with either an empty gestational sac or a gestational sac containing an embryo or fetus without fetal heart activity within the first 12 6/7 weeks of gestation, occurs in ∼20% of all clinically recognized pregnancies.^1^ Recurrent pregnancy loss (RPL) is defined by ≥ 2 failed clinical pregnancies^2^, ^3^ and affects 2%–4% of couples.^4^ It is well established that chromosomal anomalies account for 50%–70% of EPL^5^, ^6^, ^7^, ^8^ but even in RPL, random aneuploidy is the single most common etiology, accounting for >50% of cases.^5^, ^6^, ^7^, ^8^, ^9^ While commonly considered, parental *balanced* chromosomal rearrangement (such as translocations) leading to *unbalanced* rearrangements as a cause of pregnancy loss, are detected in only about 4% of RPLs.^4^, ^10^, ^11^, ^12^ Cases of RPL with no chromosomal anomalies may be due to maternal conditions such as autoimmune or endocrine diseases congenital or acquired uterine cavity defects, etc. Understanding the etiology of RPL can potentially guide future management to achieve a successful pregnancy.

The RPL workup recommended by the American Society for Reproductive Medicine (ASRM) includes parental karyotyping; screening for lupus anticoagulant, anticardiolipin antibodies, and anti‐β2 glycoprotein; uterine cavity assessment; and screening for thyroid or prolactin abnormalities.^2^ However, given that most RPL are due to random aneuploidy, it is not surprising that in most cases no cause is detected.^13^ It has therefore been suggested that chromosomal analysis in *products of conception* (POCs) should be used to guide RPL workup. If this demonstrates a random aneuploidy, further testing is obviated, which may result in cost‐savings.^13^, ^14^, ^15^ It was recently reported that combining the ASRM work‐up with POC microarray would lead to identification of an explanation for the miscarriage in 91.8% of RPL cases.^16^ Regrettably, the availability of POC has recently declined due to medical management of miscarriage which has superseded surgical evacuation.

In a previous study we have shown that maternal‐plasma genome‐wide cell‐free DNA (cfDNA)—based testing can reliably detect chromosomal anomalies in random EPL and RPL.^8^ We evaluated 86 patients experiencing pregnancy loss <14 weeks’ gestation with non‐mosaic cytogenetic results in POCs and available cfDNA results. Of these, 55 (63.9%) had a chromosomal anomaly. cfDNA‐based testing had a sensitivity of 81.8% (45/55) and specificity of 90.3% (28/31). We concluded that cfDNA‐based testing can serve as an alternative to cytogenetic analysis of POCs in guiding further management in cases of RPL: if cfDNA in the second and subsequent RPL demonstrates aneuploidy, no further action is taken; if a large unbalanced reciprocal rearrangements (>7–10Mb) is found, parental karyotyping is recommended; if no abnormality is detected, the recommended RPL workup is performed.

The purpose of the current study was to analyze the cost‐effectiveness of this approach. We recognize that costs and reimbursement vary in different health systems world‐wide. We therefore share our algorithm to facilitate local cost‐effectiveness analysis based on prevailing billing schemes and costs.

## 2 MATERIALS AND METHODS

### 2.1 Analysis design

A decision‐analytic model was developed in Microsoft Excel to compare the clinical and economic outcomes associated with various clinical evaluation pathways for RPLs. The goal of all testing pathways was to identify a possible etiology for RPL that can be used to guide further workup and clinical management.

### 2.2 Four testing pathways were compared

1) *Current ASRM RPL workup* pathway (Figure S1a): All RPL cases receive parental karyotyping; screening for lupus anticoagulant, anticardiolipin antibodies, and anti‐β2 glycoprotein; uterine cavity assessment; and screening for thyroid or prolactin abnormalities, in parallel.
2) *POC microarray pathway* (Figure S1b): All RPL cases undergo POC collection and chromosomal microarray analysis. POC is commonly collected via dilation and curettage (D&C) and rarely via chorionic villi sampling (CVS), as it requires access to a specialist and is highly dependent on patient preference. Therefore, CVS is not considered in the analysis. Euploid cases receive the recommended ASRM RPL workup without parental karyotyping. Cases with fetal large unbalanced rearrangements (>7–10Mb) receive parental karyotyping, without any other extensive RPL work‐up. Cases with aneuploidy receive no further workup.
3) *POC karyotyping pathway* (Figure S1c): This pathway is similar to the *POC microarray pathway*, except that POC chromosomal analysis is performed by karyotyping. In this scenario, cases with fetal unbalanced rearrangement due to Robertsonian translocations or large reciprocal rearrangements (>7–10Mb usually) can be differentiated from random aneuploidy and would receive parental karyotyping.
4) *cfDNA pathway* (Figure S1d): All RPL patients undergo blood sampling for genome‐wide cfDNA testing. As in the POC microarray/karyotyping pathway, cases with no aneuploidy receive the recommended ASRM RPL workup without parental karyotyping. Parental karyotyping is performed only in cases with large unbalanced rearrangements (>7–10Mb). Aneuploid cases receive no further workup.

Due to the limited accessibility of biopsy samples for repeat testing, cases with failed microarray, karyotype, and cfDNA analysis are considered as having no result and receive the recommended ASRM RPL workup.

### 2.3 Inputs

The analysis was conducted based on a hypothetical cohort of 100 RPL cases in the USA. Based on our previous clinical study^8^ and other publications, we assume that among all RPL cases, 65% are due to a fetal chromosomal anomaly, 30% are due to other etiologies that are detectable by the ASRM workup (immunologic, uterine, hormonal), and 5% are unexplained.^5^, ^6^, ^7^, ^8^, ^13^ Within the 65% cases with chromosomal anomaly, 62% of all RPL cases were attributed to random chromosomal anomalies (such as trisomies, monosomies, polyploidies), 1% of all RPL cases to fetal unbalanced Robertsonian translocations,^7^ and 2% to other fetal unbalanced rearrangements.^7^ Cases with identified fetal unbalanced rearrangements are assumed to be detectable by parental karyotyping. Of the 100 RPL cases, 33 cases would have a non‐chromosomal etiology detected by current ASRM workup. The base case analysis assumed that all RPL cases have access to medical management where a POC sample could be properly collected.

Test performances in detecting chromosomal anomalies varies depending on the sample type, sample collection method, and testing methodology. Parental karyotyping on peripheral blood is assumed to have a 100% success rate and sensitivity. The performance of microarray and karyotyping on POC samples were obtained from previous publications.^17^, ^18^ POC karyotype analysis is associated with a 32% culture failure rate^18^ and a 15% rate of maternal cell contamination (MCC),^19^ which may lead to false negative results. The remaining 53% of cases are assumed to receive karyotyping results that match the genomic makeup of the feto‐placental unit. The adjusted POC sensitivity was calculated to be 78% (53/68): the numerator represented the 53% of cases with karyotyping results matching the genomic makeup of the feto‐placental unit; the denominator represented the 68% successful cases. Using microarray analysis of POCs obviates the need for cell culture and improves the success rate to 95%.^18^, ^20^ Specificity was assumed at 100% for POC karyotyping and microarray analysis. A sensitivity of 82% and a specificity of 90% for cfDNA testing were obtained from our previous clinical study.^8^ Input variables are presented in Table 1.

**TABLE 1:** Inputs of performance for POC testing and cfDNA testing

	POC karyotyping	POC microarray	CFDNA
Failure rate	32%^18^	5%^18^, ^20^	1%^8^
Sensitivity for chromosomal anomalies	78%^18^, ^19^ ^,^ ^a^	100%	82%^8^
Able to differentiate fetal unbalanced Robertsonian translocation from free trisomies	Yes	No	No

Abbreviation: cfDNA, cell‐free DNA; POC, products of conception.

^a^ The adjusted POC senstivity was calculated to be 78% (53/68): the numerator was calculated as the number of successful cases (68) minus the number of MCC (15), 53; the denominator was the number of successful cases.

The direct costs of sample collection, chromosomal analysis and other non‐genetic tests were considered. Two blood draws were included for parental karyotyping. Costs related to additional clinical consultation or downstream clinical management were not considered in this analysis. Unit costs were assigned based on CMS clinical laboratory fee schedule rates.^20^, ^21^ Detailed unit costs inputs are presented in Table 2. Since the model time horizon was less than 1 year, no discount was applied.

**TABLE 2:** Cost inputs

Cost items	Costs^8^	HCPCS code or reference
Sample collection		
Blood draw	$3	36,415
CVS	$620	59015, 78946
D&C	$2361	59,812
Tests		
Karyotyping of blood sample	$355	88230, 88245, 88280
Karyotyping of POC sample from D&C	$338	88233, 88267, 88280
Karyotyping of POC sample from CVS	$336	88235, 88267, 88280
CMA	$1,160	81,229
cfDNA	$777	Assume the average of NIPT 81420/81507
Non‐genetic workup^a^	$1,490	Popescu et al., 2018^9^

Abbreviations: CVS, chorionic villi sampling; D&C, dilation and curettage; POC, products of conception.

^a^ Non‐genetic work‐up includes uterine anatomy imaging, test for lupus anticoagulant, test for anticardiolipin, test for anti‐beta glycoprotein antibodies, test for thyroid function, test for HbA1c, test for prolactin. The cost was estimated based on self‐pay.

### 2.4 Outcomes

In the analysis, the cfDNA testing pathway was compared against the other testing pathways. Clinical outcomes included the number of RPL cases with identified etiology (i.e., perceived benefit) through POC chromosome analysis and ASRM workup. The number of ASRM RPL workups conducted was also reported. Economic outcomes included the costs by category, total cost of each RPL testing pathway, and cost per RPL tested.

### 2.5 Evaluating uncertainty

Assumptions were made in the base case analysis to simplify potential variations present in real world practice, wherein test performance and practice patterns may differ. Hence, sensitivity analyses were conducted to address alternative test performance estimates and testing pathways. In some RPL cases, the miscarriage could be due to more than one etiology^10^, ^13^ A scenario analysis was conducted to compare the diagnostic yield across various testing pathways assuming alternative etiology distribution as reported in study by Popescu and colleagues.^13^ In real‐world practice, RPL cases may receive medical management instead of D&C. In the previous clinical study, 55% of the cases were medically managed, received CVS only for POC collection.^8^ In some clinical practice, self‐collection kit is used for POC collection on at home. However, the success rate tended to the lower than surgical management (84% vs. 100%).^22^ Scenario analysis was conducted to test for the impact of alternative POC collection method.

## 3 RESULTS

### 3.1 Comparing the American Society for Reproductive Medicine workup to the cfDNA pathway

In our hypothetical cohort of 100 RPL cases, the use of cfDNA testing to guide further workup would significantly reduce the number of cases requiring the full ASRM workup from 100 to 44. Despite this, the cfDNA pathway identified the causative etiology in 78 cases, which was substantially higher than the 33 cases identified using the ASRM workup. (Figure 2 and Table S1) Moreover, this would result in a significant cost‐savings: The total cost for a cohort of 100 cases using the *ASRM workup pathway* is estimated at $221,200, the *cfDNA pathway* would require $145,877. While the cfDNA pathway would lead to costs of $77,700 for cfDNA testing, it would also result in savings of $81,123 in unwarranted workup, such as parental karyotyping. This would result in a net cost saving of $74,723.

**FIGURE 1:**
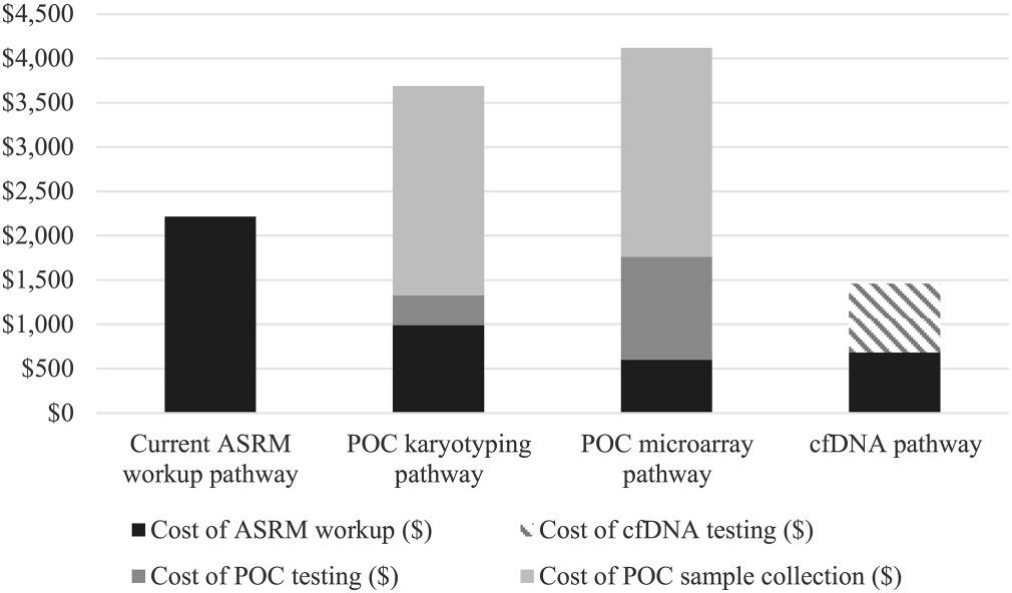
Cost of testing per recurrent pregnancy loss (RPL) case

### 3.2 Comparing the products of conception karyotype to the cfDNA pathway

The *POC karyotyping pathway* resulted in 66 cases undergoing the ASRM workup and 64 cases with a causative etiology identified (Figure 1 and Table S1). Total costs for *POC karyotyping pathway* is $368,670, which is $223,793 more than the *cfDNA pathway*.

### 3.3 Comparing the products of conception microarray analysis to the cfDNA pathway

**FIGURE 2:**
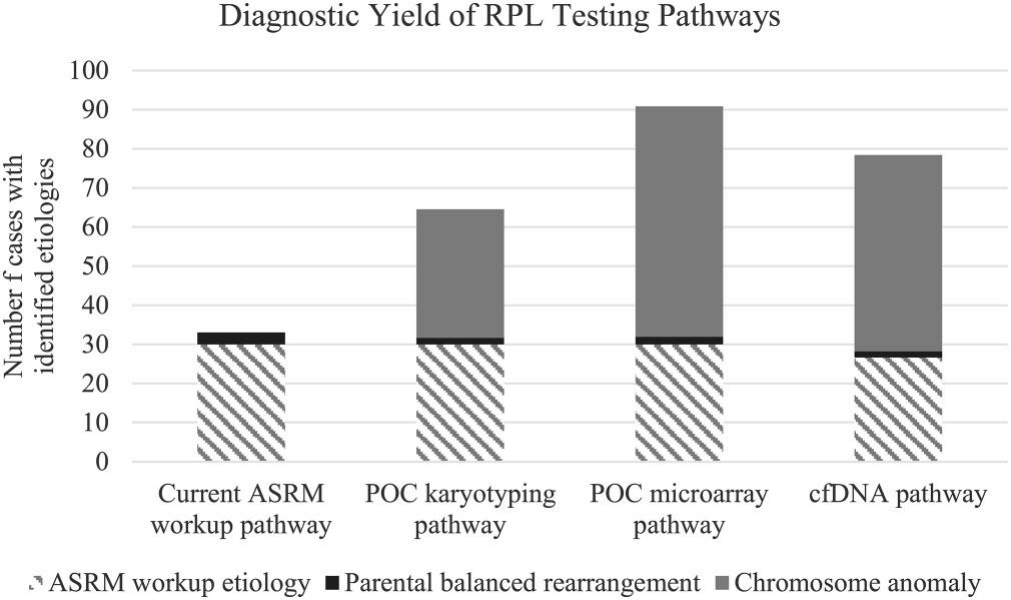
Diagnostic Yields

The *POC microarray pathway* led to the lowest number of cases requiring the full ASRM workup (39 cases) and had the greatest number of cases with an identified etiology (91). (Figure 2 and Table S1) The total cost of the *POC microarray pathway* is $411,868, which is much higher than the *cfDNA pathway*. The average cost per RPL case analyzed is $4,119, whereas the average cost per case in the *cfDNA pathway* was $1459 (Figure 1 and Table S2).

### 3.4 Scenario analysis

Scenario analyses were conducted with alternative failure rate for cytogenetic culture and alternative cfDNA pathway in which cfDNA negative cases would receive karyotyping for POC samples. In both scenarios, the total cost of the cfDNA pathway remained lower than the costs of the other RPL testing pathways (Tables S3 and S4). In the alternative etiology distribution scenario, POC pathways and the cfDNA pathway would identify at least one probable cause for the vast majority of cases. The ASRM workup pathway would identify more cases with the included etiologies than the cfDNA pathway and POC pathway (Table S5). Scenario analyses with an alternative POC collection method showed that cfDNA pathway remained cost saving in comparison to POC pathway even when only a proportion of RPL cases were surgically managed (Table S6).

## 4 DISCUSSION

This study demonstrates that using cfDNA testing to guide further workup significantly reduces the need for the complete ASRM workup, improves the proportion of RPL cases with an identified etiology compared with the current *ASRM workup pathway*, and leads to lower total cost. In comparison to the *ASRM workup pathway*, the main cost saving was achieved through the reduction in unnecessary workup driven by an aneuploidy finding. In missed abortion cases where POC have not been expelled, *POC the microarray testing pathway* could result in slightly better etiology identification than *cfDNA pathway*. However, cfDNA leads to lower total cost by the avoidance of a costly invasive procedure via uterine evacuation for POC collection. *POC karyotyping pathway* is more costly and results in fewer cases with an identified etiology and is therefore inferior to the *cfDNA pathway*.

The clinical results of comparator arms were validated against previously published studies. A retrospective cohort study was conducted by Maisenbacher et al., to evaluate the effectiveness of POC microarray and reported that microarray identified 59% of abnormal results.^23^ Papas et al., reported that combining the ASRM work‐up with POC microarray would lead to identification of an explanation for miscarriage in 91.8% of RPL cases. Both reports were highly consistent with our analysis.^16^

The analysis assumed that POC sample collection would be done in all RPL cases. However, POC sample is often unavailable due to increased utilization of medical management. In such a scenario the advantage of cfDNA testing would increase by further reduction in unnecessary ASRM workup. As technologies evolve, cfDNA is expected to reach even higher resolutions and potentially be able to identify RPL cases with microdeletion <7–10Mb, however the contribution made by such microdeletions is relatively small.^18^ This will further eliminate the need of unnecessary ASRM workup and expand the potential economic benefit. The cost of cfDNA is also expected to decrease over time. While our assumptions were based on current clinical lab fee schedule for cfDNA in the United States, 777 USD, which represent rates paid by private payers as reported to the CMS, the patient out‐of‐pocket price is usually lower. Lower cfDNA cost will lead to even greater savings in comparison to all existing pathways.

Nonetheless, this approach has limitations. The POC microarray pathway may identify the causative etiology in more cases (91) than the cfDNA pathway (79). The difference is mainly driven by identification of chromosomal anomalies that cfDNA is unable or fails to detect (such as polyploidy or small copy number variants <7Mb). Polyploidy cases, occurring in 4∼7% of RPL cases,^24^ can be diagnosed with cytogenetic culture and single‐nuleotide polymorphism (SNP) array. It can also be identified with SNP‐based cfDNA testing. Copy number variants <7Mb can only be captured by CMA. Though all cfDNA negative cases would receive the ASRM workup, the true etiology of these cases with polyploidy and small copy number variants would be missed.

The number of cases with an identified etiology revealed by the ASRM workup are expected to be similar between the POC microarray pathway and cfDNA pathway, 30 and 27, respectively. Another limitation of this study is that POC samples from spontaneous expulsion were not considered in the analysis. These samples are subject to a high rate of contamination and are often either unavailable or not accepted by clinical laboratories for RPL testing. In settings where POC samples from spontaneous expulsion are accepted, a higher rate of test failures is anticipated, which may lead to an increase in the number of cases directed to the ASRM workup. The analysis also assumes that cases with a chromosome anomaly will not receive the ASRM workup. However, it is possible in the real‐world that an RPL case would have multiple etiologies.^10^ Having POC testing or cfDNA testing as the first‐line test prior to ASRM workup may result in missed etiologies. CfDNA testing was reported to have a specificity of 90% and would lead to small fraction of false positive cases. Those cases may miss ASRM workup resulting in true etiology remaining unidentified. This caveat needs to be clearly communicated with the patient.

In the future, a prospective study should be conducted to evaluate the clinical and economic impact of implementation of cfDNA in the RPL diagnostic pathway in a real‐world setting. It will also be important to understand the performance of cfDNA testing at different gestational ages. The previous clinical study showed that the sensitivity of cfDNA test remained at a high level among RPL cases with intact sac across the gestational age ranging from 5 to greater than 9 weeks. (Figure S2). A more robust study with great sample size will help physicians determine the patient cohort most likely to benefit from cfDNA testing. Future studies should also be conducted to understand the patient acceptability and potential barriers for clinical implementation.

## 5 CONCLUSION

Our study showed that cfDNA testing was a cost‐effective strategy for RPL that led to economic saving by reducing the number of cases requiring RPL workup without increasing the number of procedures required for POC sample collection while maintaining an acceptable diagnostic yield.

## CONFLICT OF INTEREST STATEMENT

Siyang Peng and Sucheta Bhatt are employees of Illumina, Inc. manufacturer of cell free DNA testing Yuval Yaron is a member of Illumina Clinical Expert panel Antoni Borrell has no conflict of interest.

## FUNDING STATEMENT

The authors received no financial support for the research, authorship, and publication of this article.

## ETHICS STATEMENT

The study does not involve human sample nor patient information. The study also does not involve medical research in real‐world clinical setting. Therefore, it is exempted from ethics committee review.

## Supporting information

Supplementary Material S1

Supplementary Material S2

Supplementary Material S3

## Data Availability

The algorithm we developed is provided to the readers with current clinical lab fee schedule from the United States used as defaults which can be overwritten using local costs or estimates.
