# Supplementary figures and images for: Economic impact of using maternal plasma cell‐free DNA testing to guide further workup in recurrent pregnancy loss
Source: Prenat Diagn. 2021 May 24;41(10):1215–21. doi: 10.1002/pd.5972 (PMC8518071; doi:10.1002/pd.5972)

**A**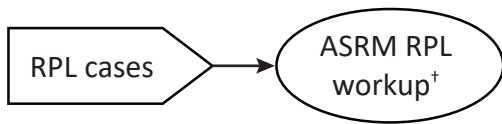**B**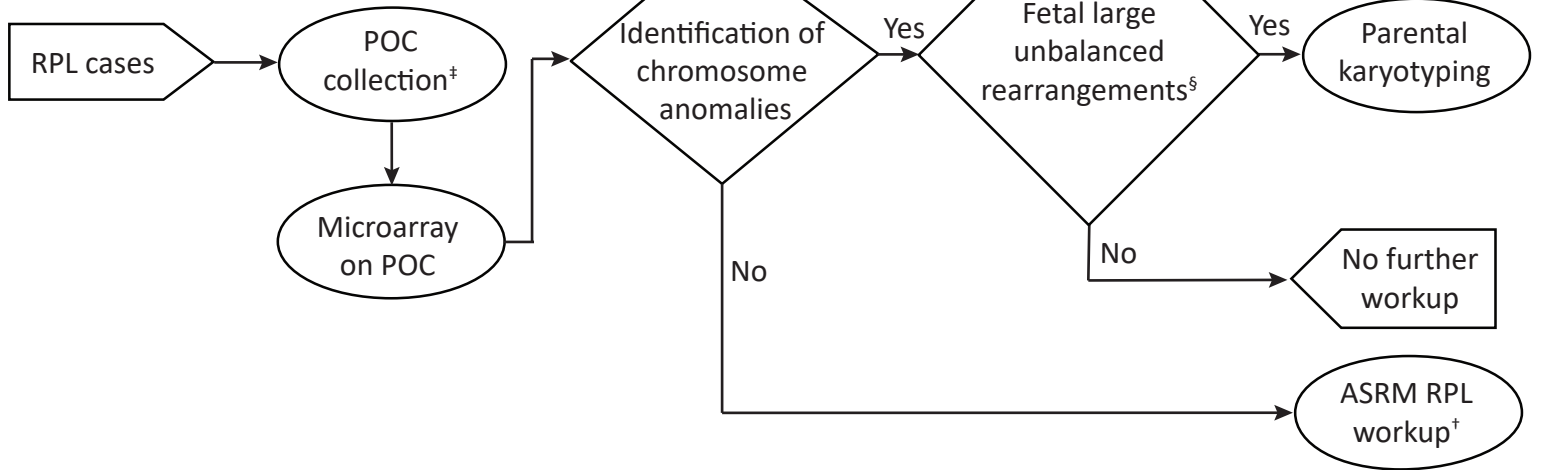**C**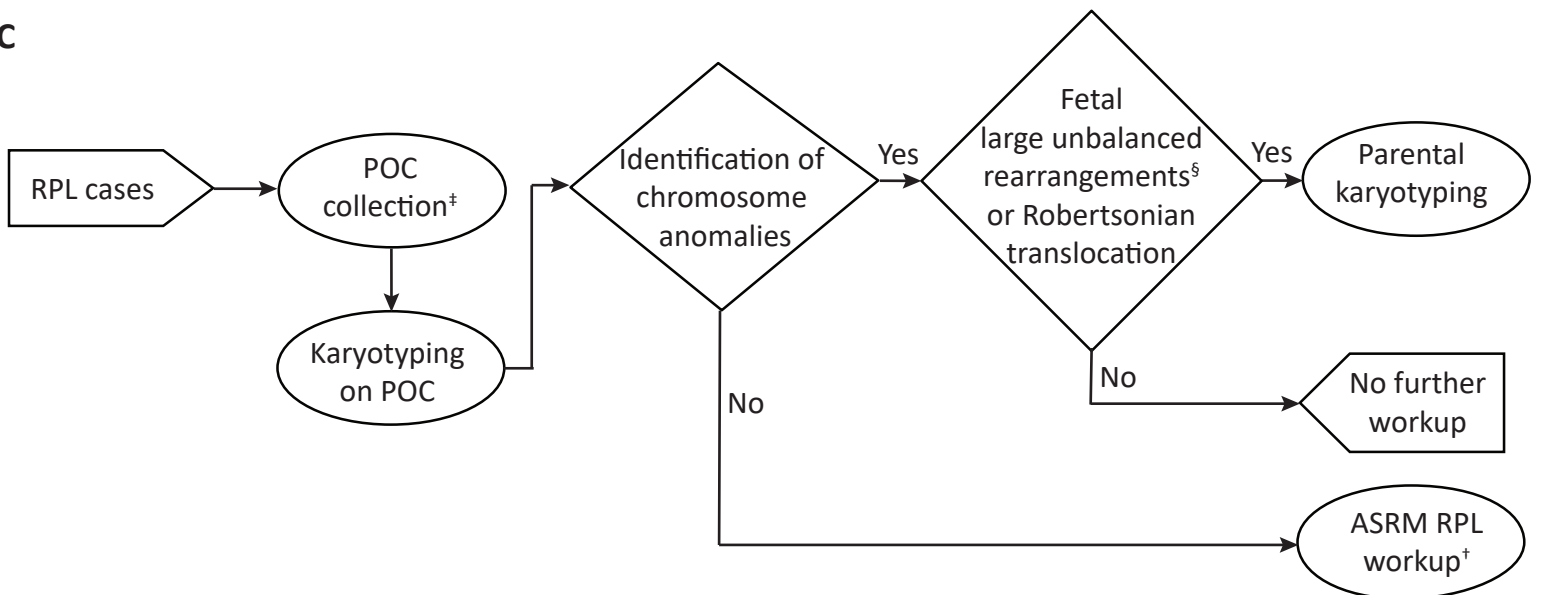**D**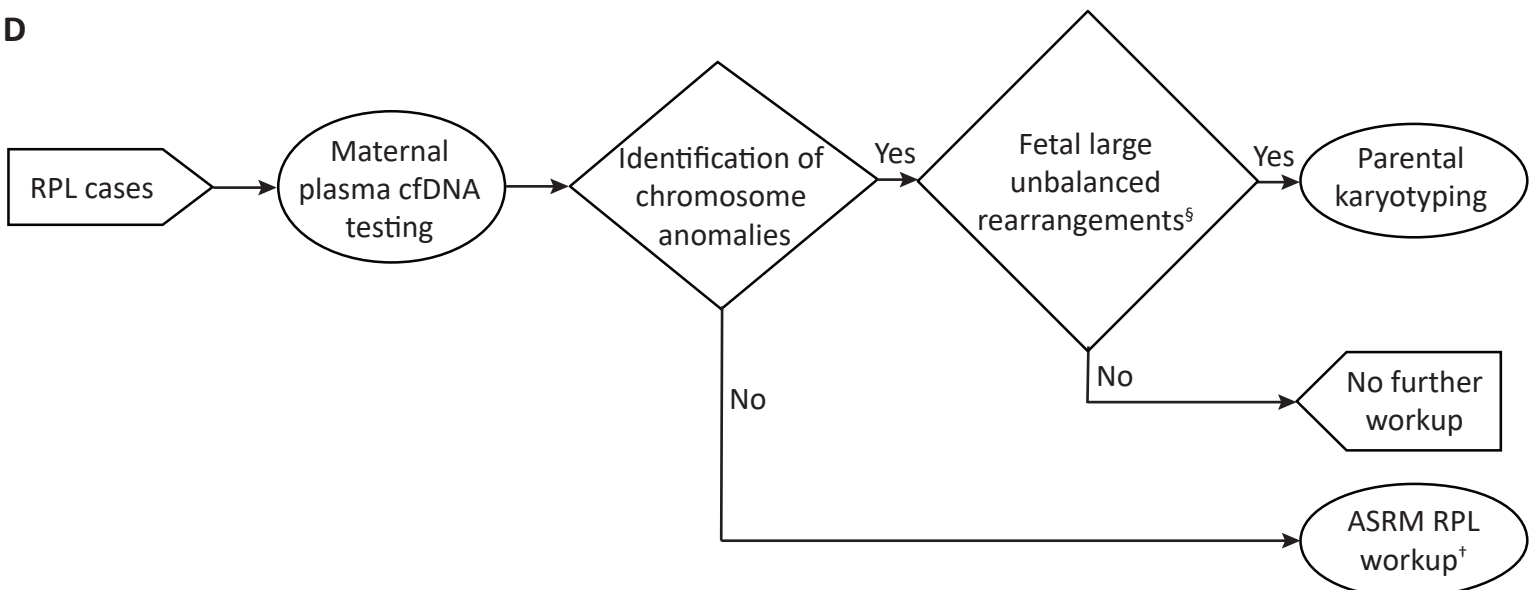

Supplement: Supplementary file 1 — Supplementary Material S1 [file PD-41-1215-s001.pdf]
