# Supplementary material for: Economic impact of using maternal plasma cell‐free DNA testing to guide further workup in recurrent pregnancy loss
Source: Prenat Diagn. 2021 May 24;41(10):1215–21. doi: 10.1002/pd.5972 (PMC8518071; doi:10.1002/pd.5972)

Cell-free DNA testing is cost-effective in recurrent pregnancy loss

Figure S2a. Sensitivity of cfDNA by gestational age based on last menstrual period

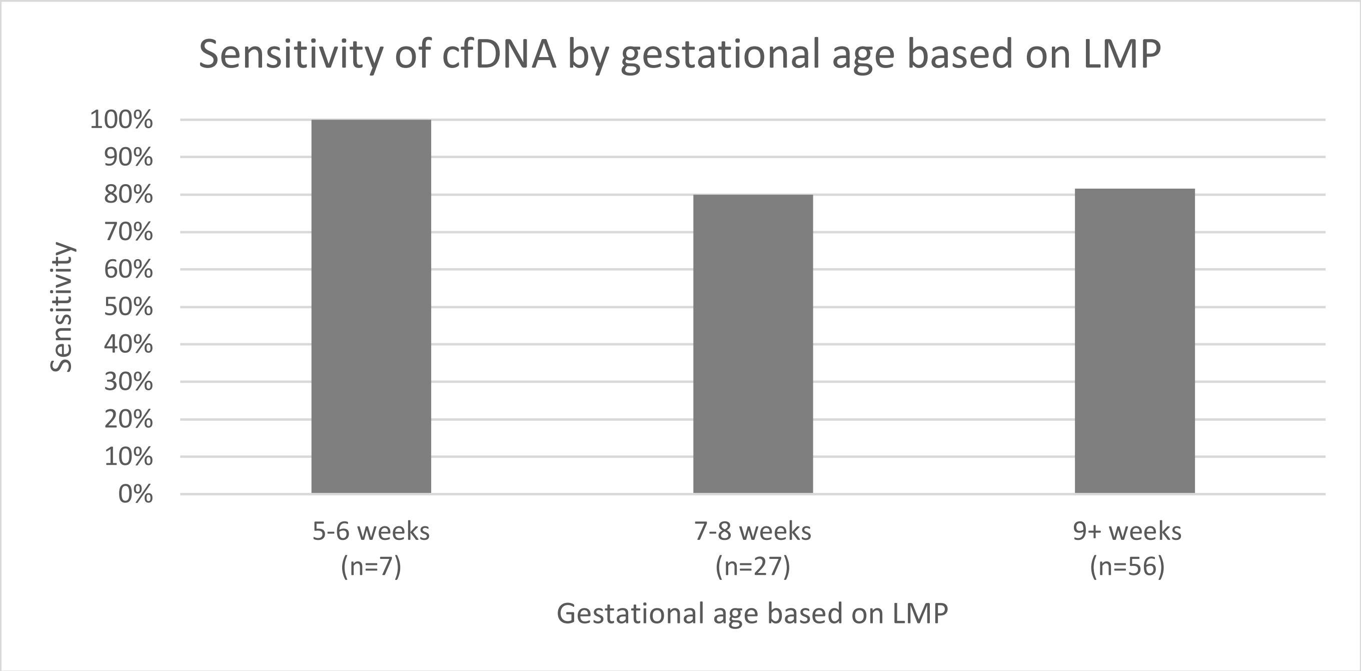

Supplement: Supplementary file 2 — Supplementary Material S2 [file PD-41-1215-s002.pdf]
